# Spinal Intradural Intramedullary Mature Cystic Teratoma in a Young Adult: A Rare Tumor

**DOI:** 10.1155/2022/9365719

**Published:** 2022-01-25

**Authors:** Aayush Shrestha, Binod Bijukachhe, Javed Ahmad Khan, Ram Krishna Dahal, Sandip Kumar Yadav

**Affiliations:** Department of Orthopedics, Grande International Hospital, Kathmandu, Nepal

## Abstract

Intradural mature teratomas are spinal tumors containing all the germinal layers and rarely present in adulthood. This study describes an unusual case of intradural mature teratoma in a 19-year-old male who presented with persistent lower limb pain and difficulty in micturition. The magnetic resonance imaging (MRI) scan showed heterogeneously enhancing intramedullary mass in the L3-L4 vertebral region and was associated with tethering of the spinal cord. Scalloping of the posterior aspect of vertebral body and narrowing of the pedicles were present. Subtotal excision of the tumor was done because of its adherence to the conus. Attempt to completely excise such adherent intramedullary tumors can lead to permanent neurological deficits. The tissue was sent for histopathological examination which showed tissue from all the three germinal layers confirming the diagnosis. The patient showed improvement of symptoms following the surgery. This study also compared the literature of similar cases and the treatments available for this disease.

## 1. Introduction

Teratomas are tumors containing tissue from all the three germ layers and result from the ectopic growth of these tissues. The occurrence of these tumors in the spinal cord in adults is extremely rare [1, 2]. Excluding the sacrococcygeal region, the incidence of teratomas is less than 0.5% of the spinal tumors [3]. We present a rare case of an intramedullary spinal cord teratoma in a 19-year-old male with its relevant review of the literature regarding similar cases.

## 2. Case Report

A 19-year-old male presented with a history of difficulty in micturition and constipation for two years and progressively increasing right thigh pain without any back pain for 6 weeks. The pain was dull aching and constant without any aggravating and relieving factors. He had been consulting urologists for the problem with micturition for 2 years. The physical examinations revealed intact sensory and motor function of bilateral lower limbs but reduced perianal sensation was present along with normal anal tone. There was no obvious deformity in the spine and no cutaneous abnormalities. The lower limb deep tendon reflexes were diminished. The routine laboratory examinations were normal.

**Figure 1:**
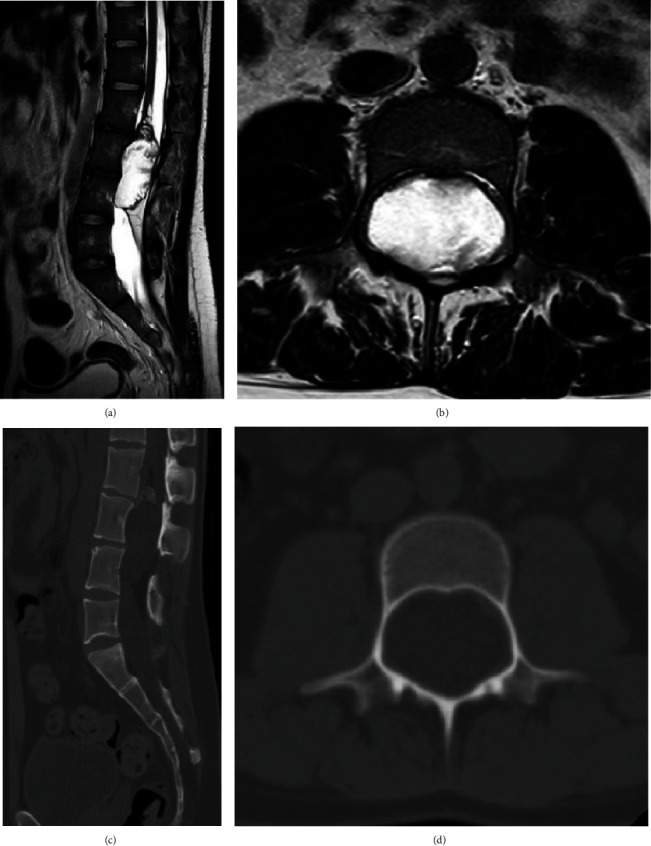
(a) Sagittal T2 MRI image showing hyperintense lesion with scalloping of the posterior aspect of the L3 vertebra. (b) Axial T2 MRI image showing heterogeneous hyperintense lesion causing expansion of the vertebral canal and narrowing of the pedicle bilaterally. (c) Midline sagittal CT image showing scalloping of the vertebra with lesions suggesting calcification at the top of the expansile lesion in the L3 level. (d) Axial CT image at the L3 vertebra level reinforcing the evidence of narrowing of the pedicles bilaterally, scalloping of the posterior vertebral body, and expansion of the vertebral canal.

The magnetic resonance imaging (MRI) revealed a well-defined lobulated, intramedullary heterogeneously enhancing mass 5 cm × 3 cm × 3.3 cm at the L3-L4 vertebral levels, suggestive of being predominantly cystic. The lesion showed components consistent with fat predominantly in the peripheral aspect. The intramedullary fat component was also seen in the conus in the superior aspect of the lesion. A mild peripheral postcontrast enhancement was seen. The spinal cord was found to be low lying terminating at the L2-L3 level due to tethering. The cauda equina nerve roots were displaced laterally by the mass lesion. Posterior element defects were present in the L5 level and proximal part of the sacrum (Figures 1(a) and 1(b)). The CT scan of the lesion was leading to the scalloping of the posterior surface of the L3 and L4 vertebra as well as thinning of the pedicles in both levels (Figures 1(c) and 1(d)).

**Figure 2:**
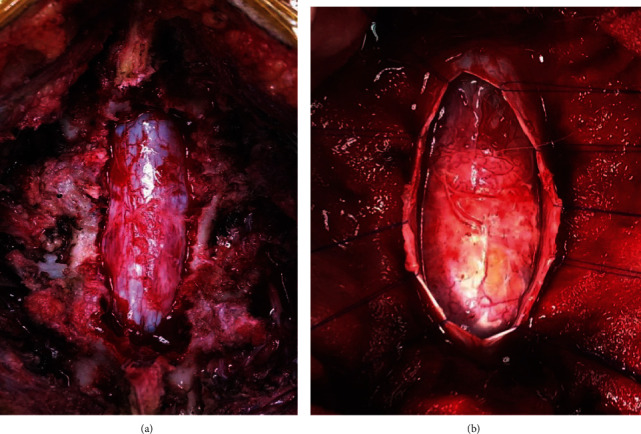
(a) Expansion of the dura seen after laminectomy at the L3-L4 level. (b) Cystic lesion evident after durotomy was done.

**Figure 3:**
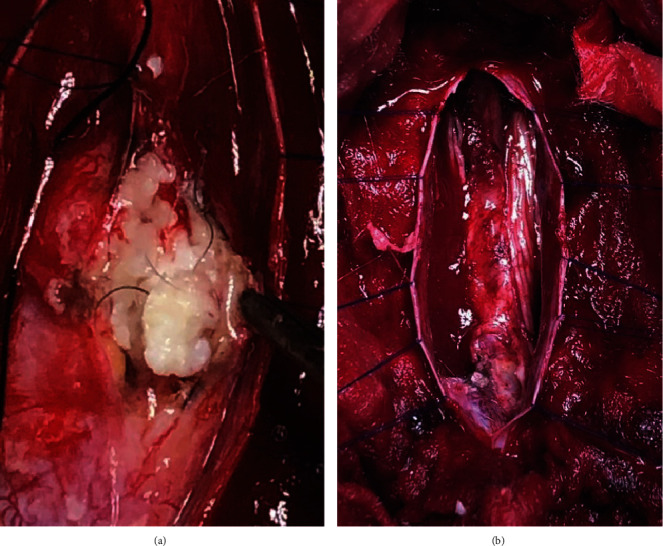
(a) Soft pearly white amorphous tissue along with hair follicles was found after the tumor was incised. (b) After subtotal excision of the tumor while preserving neurological tissues as much as possible.

The patient underwent laminectomy from L2 to L5 level and exploration of the spinal cord tumor. Intraoperatively, a distended dura was found after laminectomy. The dura was dissected, and the ill-defined cystic mass was found with a membrane 2-3 mm thick (Figures 2(a) and 2(b)). The tumor consisted of whitish amorphous heterogenous keratinaceous material along with hair follicles which were evacuated and sent for histological as well as microbiological analysis (Figure 3(a)). The proximal portion of the tumor was adherently attached to the conus so the decision was made to leave the small adherent portion to avoid severe neurological deficit which could occur if an aggressive attempt of complete excision was done (Figure 3(b)).

**Figure 4:**
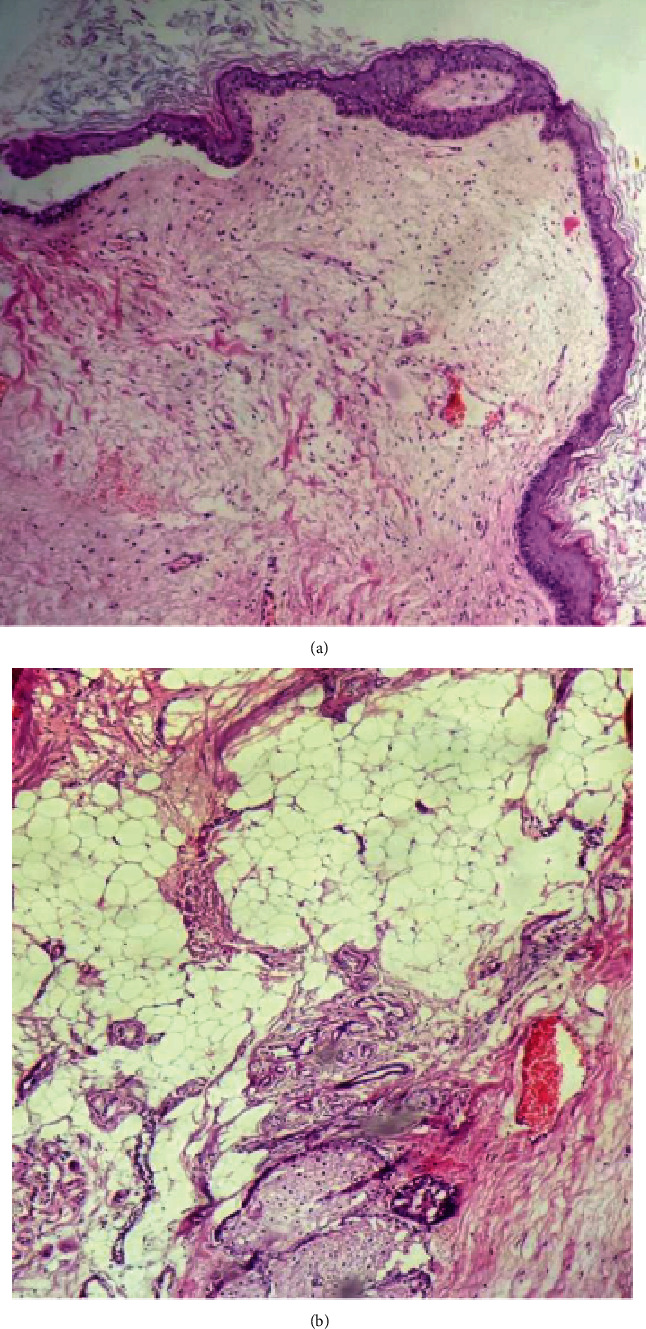
(a) Sections show keratinized stratified squamous epithelium with adnexal structures. (b) Mature fatty tissue with adnexal structures (5x magnification).

**Figure 5:**
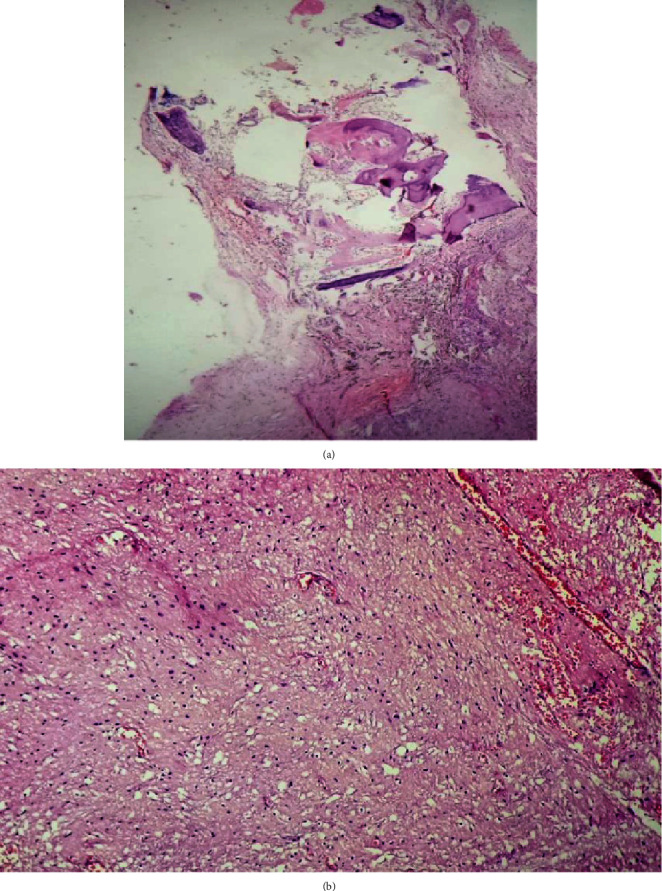
(a) Bony tissue (5x magnification). (b) Glial tissue under (20x magnification).

Histopathological examination of the mass revealed keratinized stratified squamous epithelium along with adnexal structures as well as mature fatty tissue, bony tissue without evidence of obvious malignant components (Figures 4(a), 4(b), 5(a), and 5(b)).

The final histopathological diagnosis was that of a mature cystic teratoma. The postoperative period was uneventful, and the patient remained stable.

## 3. Follow-Up

The thigh pain was relieved following the surgery while the bowel and bladder function improved after six months of surgery. A repeat MRI scan done one year after surgery showed no evidence of recurrence.

## 4. Discussion

Teratomas of the central nervous system are very rare and account for about 2% of all teratomas while their occurrence in the spinal cord is less common compared to intracranial teratomas. Approximately 0.1–0.5% of all spinal cord tumors are found to be teratomas and their occurrence in adults is even rarer [1, 4, 5]. It is more common in children and often associated with spinal dysraphism [6]. Iatrogenic cysts may be caused by the inclusion of some dermal or epidermal tissue during the closure of myelomeningocele or by the introduction of the epithelium during epidural spinal injections [7].

The clinical presentation of adults with spinal teratomas are highly variable depending upon the level of location of the tumor in the spinal column (cervical, thoracic, or lumbosacral) and whether the tumor is intramedullary, intradural extramedullary, or extradural [8]. The presenting signs and symptoms vary from motor dysfunction, sphincter disturbance, pain, sensory deficits, and disturbance in the reflexes [9]. Mature intraspinal teratomas in adults typically present with slow onset as a more localized lesion usually located between the lower thoracic and conus medullaris [10].

It is difficult to differentiate teratomas from other tumors of the spinal cord based only on imaging studies. Computed tomographic studies may show calcifications or differences in the densities within the tumor [1]. MRI is usually the preferred imaging modality for identifying these spinal tumors. They may appear as cystic or lobulated masses with variable signal intensities depending upon the cystic and solid composition. MRI is useful for localizing the tumor, determining whether it is intramedullary or extramedullary, and for preoperative planning [11].

The treatment for symptomatic patients with teratoma is surgical resection. Laminectomy for exposure and removal of the tumor and spinal decompression is done. The goal of surgery is to decompress the neural elements without causing additional damage to the normal neural parenchyma. Histopathological examination after removal of the tumor is necessary for the definitive diagnosis of the teratomas [12]. The identification of the presence of tissue representative of the three germinal layers(ectoderm, mesoderm, and endoderm) is characteristic of teratoma [13]. In several studies, only two of the three germinal layers were found, in which derivatives of one or two layers had grown over the other; therefore, the presence of only two layers does not rule out the diagnosis of teratomas [1]. These tumors are difficult to resect completely due to the adhesion to the surrounding tissues found in about 50% of cases [1, 14]. Removal of as much of the pathological tissue is recommended if complete removal of the tissue is not possible to preserve surrounding neural tissue since subtotal resection increases the chance of recurrence [15]. Recurrence of the tumor leading to the reappearance of symptoms takes a longer time due to the tendency of these tumors to grow slowly [15]. Total and subtotal resections seem to have similar recurrence rates (9% and 11%), respectively; thus, some authors do not recommend aggressive resection which could lead to neurological deficit [1, 5, 14]. To prevent the cystic contents from spilling into the intradural space which may lead to aseptic chemical meningitis with or without obstructive hydrocephalus, care should be taken during surgery [16–18]. For densely adherent lesions, intraoperative electrophysiologic monitoring, which signals when the dissection should be stopped, can be extremely useful in resection of the lesions, to prevent permanent neurological damage, especially with lesions in higher spinal levels [19–21]. Adjuvant chemotherapy and radiotherapy are generally not recommended for benign teratomas [22].

The misplaced germ cell theory and the dysembryogenic theory are the popular theories regarding the origin of teratomas. The dysembryonic theory is that the teratomas arise from a pleuripotent cell line which differentiates chaotically in a disturbed environment during their development [18]. According to the misplaced germ cell theory, the neural tube germ cells get misplaced during migration. Misplaced germ cells might be the likely cause in cases presenting as adult mature teratomas of the spinal cord with spinal dysraphism [12, 14].

Teratomas are classified as three major types of mature, immature, and malignant teratomas. While mature teratomas contain well-differentiated tissue, immature teratomas contain poorly differentiated nonmalignant tissue. Malignant teratomas are highly aggressive and have a poor prognosis. They present with high-level serum *α*-fetoprotein and are associated with yolk sac or endodermal sinus tissue [6, 14].

Long-term follow-up of the cases is required to rule out possible recurrence with repeated MRI imaging [10, 13, 22]. There is no definitive evidence of additional benefits of radiotherapy and chemotherapy in these tumors [10, 23, 24]

## 5. Conclusions

Intradural extramedullary teratomas are rare tumors. The definitive diagnosis is based on the intraoperative and histopathological examination. Total surgical excision is the primary treatment modality. Long-term follow-up is required to rule out the recurrence of the tumor.
